# Childbearing intentions, fertility awareness knowledge and contraceptive use among female university students in Cameroon

**DOI:** 10.1371/journal.pone.0276270

**Published:** 2022-10-17

**Authors:** Derick Akompab Akoku, Thomas Achombwom Vukugah, Mbah Abena Tihnje, Idris Bigweh Nzubepie

**Affiliations:** 1 Health Alliance International, Abidjan, Côte d’Ivoire; 2 ICAP at Columbia University, Mozambique Office, Maputo, Mozambique; 3 ICAP at Columbia University, Cameroon Office, Yaounde, Cameroon; 4 Center for Global Health Practice and Impact-HIV Project, Georgetown University, Yaounde, Cameroon; Ohio University, UNITED STATES

## Abstract

**Objectives:**

The primary objective of this study was to examine the association between fertility awareness knowledge, and contraceptive use among sexually active female university students (FUS) in Cameroon.

**Methods:**

This study was designed as a secondary data analysis of a cross-sectional survey that was conducted between July and August 2018. We extracted and analyzed relevant data (i.e., socio-demographic characteristics, sexual behavior, fertility-related characteristics, and contraceptive use) using a modified Poisson regression with a robust variance estimator. Prevalence Ratios (PR) and 95% confidence intervals were estimated, and statistical significance was set at P≤0.05.

**Results:**

The median age of the sexually active FUS was 23 years (IQR = 21–25) and 99.3% indicated that they wanted to have children. Only 49.3% knew their fertile period and 62.5% of the sexually active FUS were current contraceptive users. We found a statistically significant association between fertility awareness knowledge and period abstinence (PR = 1.57;95%CI: 1.02–2.44, p = 0.049). In multivariate adjusted models, there was a statistically significant association between fertility awareness knowledge and male condom use (APR = 1.29; 95% CI:1.02–1.64, p-value = 0.032) and the withdrawal method (APR = 1.40;95% CI:1.02–1.93, p = 0.038). We found a statistically significant effect modification of “preferred timing to have children” on the association between fertility awareness knowledge and withdrawal method use. There was no association between fertility awareness knowledge and the use of oral contraceptive pills.

**Conclusion:**

Most of the female students intend to have children in the future, but their fertility awareness knowledge was suboptimal. There was a statistically significant relationship between fertility awareness knowledge, and the use of male condoms and the withdrawal method. The study underscores the need for FUS to be targeted with interventions to help them gain knowledge of their menstrual cycle to better plan or avoid unwanted pregnancy.

## Introduction

An estimated one-third of the global burden of disease and early death among women of reproductive age is attributed to their poor sexual and reproductive health [1]. In sub-Saharan Africa (SSA), many women face sexual and reproductive health challenges which affect their physical, social and psychological wellbeing [2]. In Cameroon, there is a high level of maternal mortality with about 529 per 100,000 live births recorded in 2017 and contraceptive use is relatively low in the country [3]. A national population-based survey conducted in 2018 found that only 19% of married women aged 15–49 years used any method of contraception: 15% used a modern method, and 4% used a traditional method. The use of modern contraceptive methods was higher among sexually active nonmarried women [4].

A previously conducted study in Cameroon found that about 40% of all pregnancies in the country are unintended (unwanted or mistimed), and 36% of these unintended pregnancies end in abortion [5] and unsafe abortion contributes to maternal morbidity and mortality [2]. In Cameroon and other countries in SSA, many women also face sexual and reproductive health challenges associated with the HIV epidemic, which has been reported to be among the leading cause of mortality among women aged 15–49 years in these countries [6].

There is evidence that the use of safe contraceptive methods can reduce the risk of unintended pregnancy, reduce unsafe abortion, and reduce the likelihood of contracting or transmitting HIV/STI [7–9]. Although many women are knowledgeable about contraception, the use of contraceptives has been low in many settings [10, 11]. In Cameroon and other SSA countries, uptake of contraceptives has been hindered by a number of barriers, including but not limited to: misconceptions and misinformation, fear of side effects, cost, refusal of spouse, lack of information, belief that it will increase risky sexual behaviour and for religious reasons [12–14].

Previous research has demonstrated that fertility awareness knowledge could be helpful in reducing unintended pregnancy among women [15]. Fertility awareness has been described as the ability of a woman to identify the days during her menstrual cycle when unprotected sexual intercourse is most likely to result in pregnancy, and this is usually achieved through monitoring various signs and symptoms of fertility [16]. Knowledge of the fertile period can enable the woman to modify her sexual behavior, deciding whether to either prevent unintended pregnancy or achieve pregnancy. Therefore, women may avoid unprotected sex during the fertile period or use a barrier method to prevent unintended pregnancy. A woman may also decide to have unprotected intercourse to become pregnant during her fertile period [16–18]. Despite the benefits of this strategy to prevent unintended pregnancy, most young women in SSA do not understand their menstrual cycle and fertility awareness knowledge has been reported to be low [19]. The lack of fertility awareness knowledge in the absence of or misuse of contraceptives could lead to negative health outcomes such as unintended pregnancy [15, 20].

In many settings across SSA, many young girls and women are at increased risk of unintended pregnancies [21–24] and female university students (FUS) represent a subgroup highly vulnerable to having unintended pregnancies. A review of the published literature reveals that there are limited studies that have examined fertility awareness knowledge among FUS in Cameroon. Population-based national surveys mainly targeted women in the general population, which makes it difficult to generalize such findings to FUS in the country. Consequently, little is known about the proportion of FUS in the country who know their fertile period and whether fertility awareness knowledge shapes their contraceptive behaviour and practices. This study was designed to fill the knowledge gaps and provide data that may inform the design and implementation of programs aimed at improving the reproductive health knowledge and practices of FUS.

The objectives of this study were to: (1) determine fertility desires and the factors associated with fertility awareness knowledge and (2) examine the association between fertility awareness, knowledge, and contraceptive use among sexually active FUS in Cameroon. This paper uses a subsample of data from a larger study that investigated the sexual and reproductive health of FUS in Yaounde, Cameroon.

## Conceptual framework

**Fig 1:**
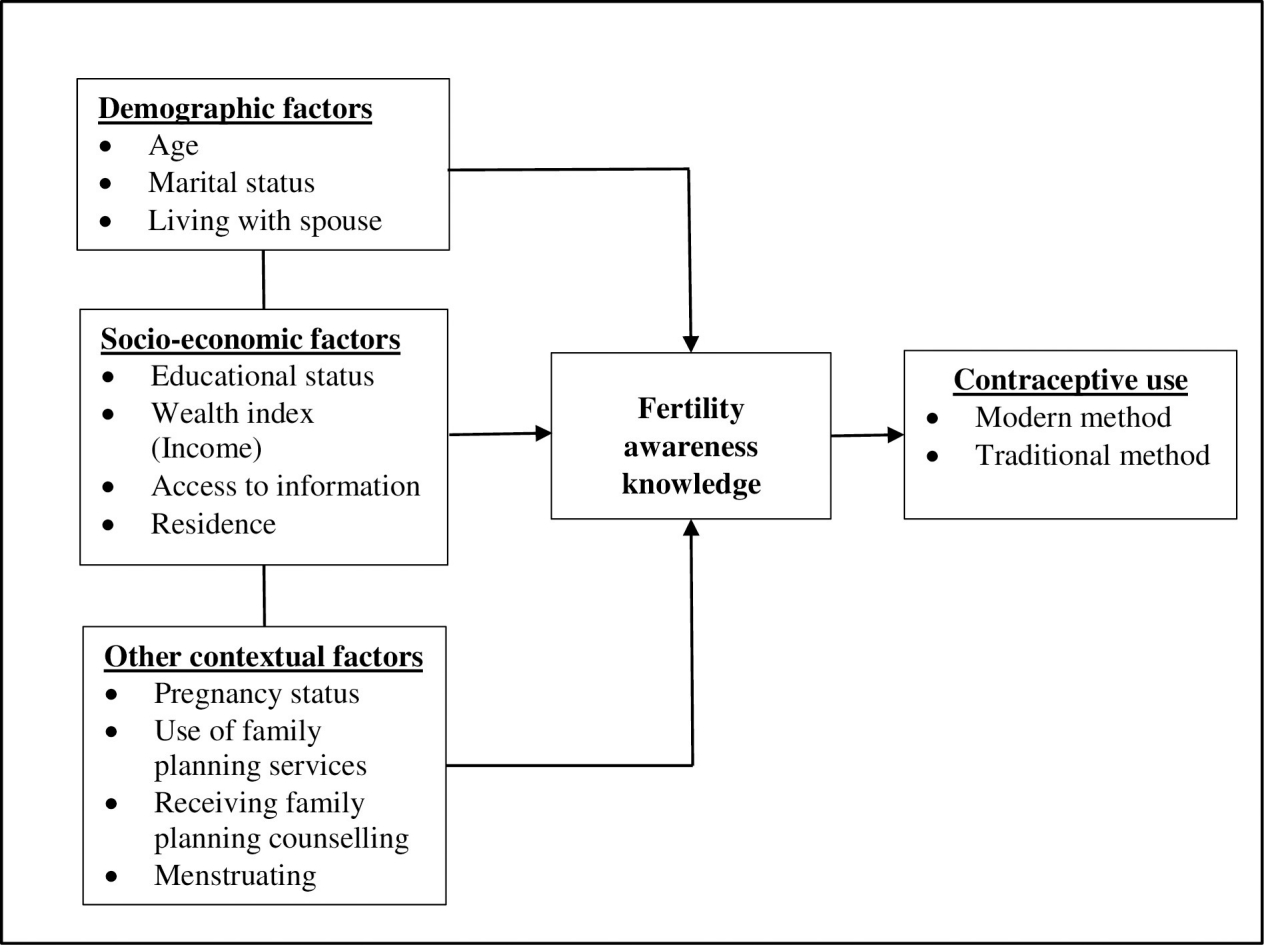
Conceptual framework for factors associated with fertility awareness knowledge among young women in sub-Saharan Africa.

The current study presents a conceptual framework based on previous studies to help understand the determinants of fertility awareness knowledge among young women (unmarried and married) in SSA (Fig 1). A review of published literature demonstrates that fertility awareness knowledge is shaped by three main factors i.e., demographic factors, socio-economic and other contextual factors. Individual-level factors mainly include older age, marital status and living with the spouse [19, 25].

The socio-economic factors include level of education, wealth index, exposure to sexual health information (through the media, school, parents, friends etc) and place of residence. Research conducted in SSA has reported that young women with a higher level of education are likely to correctly identify the timing of ovulation [15, 19]. Higher level of education increases knowledge and understanding of health information through increased access to information and health literacy [26]. Furthermore, young women in rural areas are more likely to have incorrect knowledge of their fertile period, which may be attributed to their limited access to information and other socio-cultural beliefs around issues of fertility [15]. Mass media exposure increases young women’s knowledge about sexuality and fertility, dispelling myths and misconception, and overcoming any barriers [27].

Health-related factors that have been associated with fertility awareness especially among married women in SAA include: receiving family planning counselling, use of family planning methods, being pregnant, and having a menstruation [25]. It is important to keep in mind that while the above factors promote fertility awareness, there are several cultural norms that prevent young women having good fertility awareness knowledge. In some countries, discussions related to menstruation and fertility in a family or community setting is considered a taboo and there is stigma and embarrassment which undermines young women’s knowledge and understanding of sex, ovulation and menstruation [28].

Based on past empirical evidence, the guiding hypothesis for this study was that fertility awareness knowledge will promote contraceptive use among sexually active FUS. This hypothesis was based on previous research which found that young women with correct knowledge of their fertile period are more likely to use contraceptives to reduce the risk of unintended pregnancy [15]. Although previous studies had established this relationship, this study was designed to determine which specific modern and traditional contraceptive methods commonly used by sexually active FUS were associated with fertility awareness knowledge.

## Methods

### Data source

We used data (S1 Dataset) from a Sexual and Reproductive Health Study that was conducted between July and August 2018 among FUS at the University of Yaounde 1, Cameroon. We extracted data (i.e., socio-demographic characteristics, sexual behavior, fertility-related characteristics, and contraceptive use) for the 424 sexually active FUS who participated in the parent study. In the parent study, self-administered questionnaires were used to collect data from 773 female students and the detailed methods (including design, setting, eligibility criteria, sample size, and participant recruitment) of the parent study have been published elsewhere [29].

The study protocol for the parent study was reviewed and approved by the Cameroon National Research Ethical Committee for Human Health (No: 2018/07/1084/CE/CNERSH/SP). In addition, administrative authorization was obtained from the Rector of the University of Yaounde 1 to collect data from female students at the university. All students provided written informed consent prior to study participation [29].

### Variables of interest and measures

The first objective of this study was to determine the factors associated with “fertility awareness knowledge” among the study population. Fertility awareness knowledge was the outcome variable and was defined as a student’s knowledge of the point during her menstrual cycle at which she is most likely to become pregnant after having unprotected sexual intercourse. This was assessed by asking participants: *‘From one menstrual period to the next*, *there are certain days when a woman is more likely to get pregnant if she has unprotected sex*?*’ Which of these best describes your fertile period when you are most likely to conceive if you have unprotected sex*? They were provided with 5 response choices. Participants with correct knowledge of their fertile period (“yes”) where those who answered that they are more likely to get pregnant if they practice unprotected sex halfway between their two menstrual cycles, while those with incorrect knowledge (“no”) were those who mentioned otherwise or responded that they do not know the fertile period of their menstrual cycle. This question has previously been asked to women in national Demographic and Health Surveys across countries in SSA [30, 31].

The explanatory variables included: age (<25 yrs, ≥25 yrs); marital status (never married, married), level of study (undergraduate and postgraduate), religion, awareness of HIV status, accessing reproductive health information on social media at least once in the past 3 months (no/yes), preferred number of children, preferred timing to have children and fertility tracking methods.

Awareness of HIV status was measured by asking respondents the following question: “*Have you ever been tested for HIV*?” Those who responded “yes” were further asked: “*When was the last time you had an HIV test*?” followed by “*Did you receive your test results the last time you tested for HIV*? Awareness of HIV status was treated as a binary variable and the responses were coded as “yes” or “no”. We considered those who were aware of their HIV status (“yes”) as those who were tested in the past 12 months and received their test results”. Those who were not aware of their HIV status (“no”) were considered as those who had never been tested for HIV, those who were tested for HIV more than 12 months ago, and those who were tested for HIV in the past 12 months but never received their test results [32].

The second objective of this study was to investigate the association between fertility awareness knowledge, and contraceptive use. The main outcome variables were the two most commonly used traditional and modern contraceptive methods which respondents used in the past 3 months. In the parent study, participants were asked the following question: *Have you used any contraceptive method in the past 3 months to prevent unintended pregnancy*? *(no/yes)*. Those who indicated “yes” were asked to indicate the type of contraceptive method they used. Contraceptive methods were classified into traditional and modern methods, and the prevalence of current contraceptive use was estimated by dividing the number of users by the number of sexually active students who participated in the study.

The primary exposure variable was fertility awareness knowledge (yes/no) and the covariates included age, marital status, study level, religion, awareness of HIV status, number of sexual partners (single/multiple), accessed reproductive health information on social media at least once in the past 3 months, preferred timing to have children and fertility tracking method.

### Data analysis

Data were analysed for the 424 sexually active FUS who participated in the parent study. Descriptive statistics were summarized with proportions (or percentages) for categorical data and median (inter-quartile range) for continuous data. Chi-square tests for categorical variables were used to assess bivariate associations between demographic, and fertility-related characteristics and fertility awareness knowledge.

To identify the independent factors associated with fertility awareness knowledge, variables that were significant at p<0.25 in the bivariate analyses were considered candidate variables for multivariate analysis. We checked for multicollinearity by assessing the variance inflation factors of the variables included in the adjusted model. Although the variable “childbearing intention” was significant in bivariate analysis, it was dropped from the adjusted multivariate model due to multicollinearity. We used a modified Poisson regression model with robust error variance to estimate prevalence ratio (PR) and 95% confidence intervals. We used PR because the study was cross-sectional, the outcomes were common, and the log-binomial model did not converge [33].

We further evaluated the association between fertility awareness knowledge and contraceptive use in four separate unadjusted and multivariate adjusted models. Each model included one of the four most commonly used contraceptive method (as the outcome) and fertility awareness knowledge (as the primary exposure). The outcome variable in Model 1, Model 2, Model 3, Model 4 was male condom, oral contraceptives, withdrawal, and periodic abstinence respectively. Age was modelled as a continuous variable in all the four models. Each multivariate model was adjusted for age, marital status, level of studies, religion, awareness of HIV status, knowledge of last sexual partners HIV status, accessed reproductive health information at least once in the past 3 months and preferred timing for next child.

We identified potential interactions a-priori and tested their interaction terms in the models which had “male condom use” and “withdrawal” as the outcome variable. We examined if marital status, awareness of HIV status, number of sexual partners and preferred timing to have children modified the association between fertility awareness knowledge and male condom use. We further evaluated whether marital status and preferred timing to have children modified the association between fertility awareness knowledge and the use of the withdrawal method. We performed separate full multivariate models with each of the interaction terms and later conducted a Wald test for the interaction term. We considered effect modification if the p-value of the Wald test of the interaction term was < 0.05. All statistical tests were two-tailed and were considered statistically significant at p-value≤0.05. Data were analysed using STATA version 14.0 (Stata Corp, College Station, Texas, USA).

## Results

Table 1 shows the demographic and fertility-related characteristics of participants by fertility awareness knowledge. The median age of participants was 23 years (IQR = 21–25), most of them were never married (83.2%), and 52.1% were non-Catholics. Less than half (49.3%) knew their fertile period and 54.7% were aware of their HIV status. Only 30.0% of the students reported that they accessed reproductive health information at least once on social media in the past 3 months; Facebook (59.2%) and WhatsApp (34.7%) were the most widely accessed social media platforms.

**Table 1:** Demographic & fertility related characteristics by fertility awareness knowledge.

Variables	TOTAL N (%)	Fertility awareness knowledge		P-value
		Yes n (%)	No n (%)	
**Sample size**	424 (100)	209(49.3)	215(50.7)	
**Age group (years)**				0.051
<25	279(65.8)	128(45.9)	151(54.1)	
25+	145(34.2)	81(55.9)	64(44.1)	
**Marital status**				0.602
Never married	353(83.2)	172(48.7)	181(51.3)	
Married or Divorced	71(16.8)	37(52.1)	34(47.9)	
**Level of study**				0.466
Undergraduate	319(75.2)	154(48.3)	165(51.7)	
Postgraduate	105(24.8)	55(52.4)	50(47.6)	
**Religion**				0.444
Catholic	203(47.9)	104(51.2)	99(48.8)	
Non-Catholics	221(52.1)	105(47.5)	116(52.5)	
**Awareness of HIV status**				0.271
No	192(45.3)	89(46.4)	103(53.7)	
Yes	232(54.7)	120(51.7)	112(48.3)	
**Number of sexual partners**				0.252
Single	339(80.1)	162(47.8)	177(52.2)	
Two or more (multiple)	84(19.9)	46(54.8)	38(45.2)	
**Accessed reproductive health information on social media at least once in the past 3 months** ^**1**^				0.026
No	229(70.0)	122(53.3)	107(46.7)	
Yes	98(30.0)	39(39.8)	59(60.2)	
**Childbearing Intentions**				0.087
No	3 (0.7)	0(0.0)	3(100.0)	
Yes	421(99.3)	209(49.6)	212(50.4)	
**Preferred number of children(n = 421)**				0.461
1–3	95(22.6%)	44 (46.3)	51(53.7)	
4+	326(77.4%)	165(50.6)	161(49.4)	
**Preferred timing to have children (n = 419)** ^**2**^				0.094
During studies	121(28.9)	52(42.9)	69(57.1)	
After my studies	298(71.1%)	155(52.0)	143(48.0)	
**Fertility tracking methods** ^**3**^				0.138
Calendar-based	330(77.83)	169(51.2)	161(48.8)	
Symptoms-based	94(22.17)	40(42.6)	54(57.5)	

^1^Social media platforms from which they accessed reproductive health information included Facebook (59.2%) WhatsApp (34.7%) and Others including YouTube (6.1%).

^2^The total number of responses was 419 due to missing data

^3^Symptom-based methods included temperature and cervical mucus.

Nearly all (99.3%) of the FUS indicated that they wanted to have children. Of those with childbearing intentions (n = 421), 77.4% preferred to have at least four children and 71.1% preferred to have children after their university studies. Most (75.7%) of those who wanted children after their studies were students who had been never married. About 77.8% of students used the calendar method to track their fertile period.

### Factors associated with fertility awareness knowledge

**Table 2** shows the factors associated with fertility awareness knowledge among the students. In multivariate-adjusted Poisson regression models, students aged ≥25 years were 27% times more likely to know their fertile period (APR = 1.27; 95%CI: 1.02–1.59, p-value = 0.031) compared to younger aged (<25 years) students. Those who accessed reproductive health information on social media at least once in the past 3 months were 25% times less likely to know their fertile period (APR = 0.75; 95%CI: 0.57–0.99, p-value = 0.046) compared to those who did not access reproductive health information on social media at least once in the past 3 months.

**Table 2:** Multivariable Poisson regression model assessing predictors of fertility awareness knowledge.

Variables	Adjusted Prevalence Ratio (95% CI)	P-value
**Age group (years)**		0.031
<25	1.0	
25+	1.27(1.02–1.59)	
**Accessed reproductive health information on social media at least once in past 3 months**		0.046
No	1.0	
Yes	0.75(0.57–0.99)	
**Preferred timing to have children**		0.076
No	1.0	
Yes	1.26(0.97–1.63)	
**Fertility tracking methods**		0.327
Calendar	1.0	
Symptom-based	0.86(0.64–1.16)	

**Currently used contraceptive methods.** Of the 424 sexually active FUS who participated in the study, 62.5% (265/424) were current contractive users. Table 3 shows the types of contraceptive methods used in the past 3 months prior to the study by these students. The most commonly used traditional method was the withdrawal method (28.8%), followed by periodic abstinence (16.7%). On the other hand, the most commonly used modern contraceptive method was male condoms (43.9%), followed by oral contraceptive pills (25.2%).

**Table 3:** Current contraceptive methods used by sexually active female university students (N = 424).

Contraceptive Method^1^	Frequency^2^ (N)	Percent (%)
**Traditional methods**		
Withdrawal	122	28.8%
Periodic abstinence	71	16.7%
Folkloric methods^3^	15	3.5%
**Modern methods**		
Male condom	186	43.9%
Oral contraceptive pills	107	25.2%
Female condom	14	3.3%
Injectables	13	3.1%
Lactation Amenorrhea	12	2.8%
Implant	7	1.7%
Intra Uterine Device	2	0.5%
Diaphragm	1	0.2%

Notes:

^1^An estimated 62.5% of students who participated in the study were current contraceptive users.

^2^Multiple responses were permissible

^3^Folkloric methods include drinking salt and water; drinking whisky after sex; drinking Nescafé without sugar, drinking honey mixed with quinine, and drinking aloe vera.

### Association between fertility awareness knowledge and specific contraceptive methods used in the past 3 months

Table 4 shows the association between fertility awareness knowledge and the two modern and two traditional contraceptive methods commonly used by the sexually active FUS in the past 3 months. In unadjusted models, fertility awareness knowledge was significantly associated with the use of male condoms (PR = 1.30; 95%CI:1.04–1.62, p = 0.017) and periodic abstinence (PR = 1.57;95%CI: 1.02–2.44, p = 0.049). There was also a marginally statistically significant association between fertility awareness knowledge and the use of the withdrawal method (PR = 1.33;95%CI: 0.98–1.81, p = 0.059).

**Table 4:** Association between fertility awareness knowledge and methods of contraceptive use.

Contraceptive method	Unadjusted model		Adjusted model^1^	
	PR (95% CI)	P-value	APR (95% CI)	P-value
Male condom	1.30(1.04–1.62)	0.017	1.29(1.02–1.64)	0.032
Oral contraceptive pills	1.31(0.94–1.82)	0.107	1.27(0.86–1.89)	0.223
Withdrawal	1.33(0.98–1.81)	0.059	1.40(1.02–1.93)	0.038
Periodic abstinence	1.57(1.02–2.44)	0.040	1.43(0.88–2.34)	0.146

^1^Adjusted for age, marital status, level of study, religion, awareness of HIV status, number of sexual partners (single/multiple), accessed reproductive health information on social media at least once in the past 3 months, preferred timing to have children, and fertility tracking method.

In the multivariate adjusted model, students who knew their fertile period were 29% times more likely to have used male condoms (APR = 1.29; 95% CI:1.02–1.64, p-value = 0.032) compared to those who didn’t know their fertile period. There was a significant association between fertility awareness knowledge and withdrawal method use as those who knew their fertile period were 40% more likely to have used the withdrawal method (APR = 1.40;95% CI:1.02–1.93, p = 0.038) compared to their peers who were not aware of their fertile period. There was a statistically significant effect modification of “preferred timing to have children” on the association between fertility awareness knowledge and withdrawal method use. The prevalence of withdrawal method use was higher among female students who preferred to have children after their studies compared to those who preferred to have children during their studies (Wald test p-value = 0.037). We did not find any statistically significant association between fertility awareness knowledge and the use of oral contraceptive pills.

## Discussion

To our knowledge, this is the first study in Cameroon that has examined the association between fertility awareness knowledge and specific types of contraceptives used by sexually active FUS. Our analysis found that 49.3% of the female students knew their fertile period, while the rest (50.7%) did not know when they are most likely to become pregnant if they have unprotected sexual intercourse. The fact that about half of the students did not know their fertile period represents a substantial gap and challenge as these students are at a heightened risk of becoming pregnant if they have unprotected sex during their fertile days. Our findings are consistent with that of a previously conducted study in Uganda which reported low fertility awareness knowledge (35%) among female first year university students [34].

The majority (70.9%) of respondents in our study indicated that they preferred to have children after their studies and similar results have been reported in Saudi Arabia [35]. There is evidence that the lack of fertility awareness knowledge is associated with unintended pregnancy [15]. Consequently, if the female students without knowledge of their fertile period practice unprotected sex and become pregnant during their studies, some of these pregnancies may be considered as unintentional which may likely resort to abortion [2, 36].

There was a statistically significant association between age and fertility awareness knowledge. Older sexually active students (≥25 years) were more likely to know their fertile period compared to their younger counterparts. This finding is similar studies previously conducted in the United States and Australia (although not among FUS) which reported higher fertility awareness knowledge among older young women [37, 38].

In this study, married students were more likely to know their fertile period compared to single/never married students (52.1% vs 48.7%). Although this finding is not statistically significant, it is important to highlight that married students are more likely to be older and fertility awareness knowledge increases with age [37]. It is also plausible that married students in this study may have been knowledgeable of their fertile period due to family planning conversations with their spouses [16, 39] or through family planning counselling sessions they may have attended in public and private clinics [40].

We found a significant, but negative association between access to reproductive health information at least once on social media in the past 3 months and fertility awareness knowledge. While more research is needed, this inverse association could be explained by the fact that students may have accessed reproductive health information which may not have been related to fertility. Our results are surprising despite the promising evidence that social media could be useful to help young women access sexual and reproductive health information [41, 42]. This finding suggests the need for fertility-awareness education to be provided to adolescent girls and young women via social media platforms.

The prevalence of current contraceptive use was high (62.5%) and is consistent with that of a study conducted in Tanzania, where 61% (n = 162/267) of sexually active FUS were current contraceptive users [43]. However, other studies in Tanzania [10], Uganda [24] and Nigeria [44] have reported low current contraceptive use among sexually active FUS. The withdrawal method and periodic abstinence (traditional) as well as male condoms and oral contraceptive pills (modern) were the most commonly used contraceptive methods reported by the female students. Our findings are consistent with those of previous studies conducted in other African countries which reported that the above methods were widely used by FUS [10, 45, 46].

Overall, this study found a statistically significant association between fertility awareness, knowledge and current contraceptive use. A study conducted among young women across SSA reported that fertility awareness knowledge was associated with contraceptive use [15]. In this study, we examined the association between fertility awareness knowledge and the two most commonly used traditional and modern contraceptive methods. In both the unadjusted and multivariate adjusted models, we found a statistically significant association between fertility awareness knowledge and the use of male condoms. Our results support the body of evidence that women who practise fertility-awareness-based methods use male condoms when having sexual intercourse during their fertile days to prevent unintended pregnancy [16, 18].

In our study, 28.8% of FUS used the withdrawal method and we found a statistically significant association between fertility awareness knowledge and the use of the withdrawal method. Our results are consistent with the academic literature which has reported that the withdrawal method is widely practiced by women who use fertility awareness-based methods for family planning [16]. However, women who rely on the withdrawal method must accurately know their fertile days because any mistakes may lead to unintended pregnancy [18] and this underscores the need for more reproductive health information to target FUS. The efficacy of this method is largely dependent on the male partner withdrawing before any sperm is released into the vagina and relies on the male being able to accurately identify when to withdraw and being compliant [18].

Our study did not find any significant association between fertility awareness knowledge and the use of oral contraceptive pills and similar findings had previously been reported in Australia among young women [38]. This finding is due to the fact that the use of oral contraceptive pills does not depend on fertility awareness knowledge as women can have unprotected sex throughout their menstrual cycle while taking these pills [16].

In unadjusted models, students who knew their fertile period were more likely to have practiced periodic abstinence. We found that most students who practiced periodic abstinence used the calendar-based method than the symptom-based method (18.4% vs 10.6%) to track their fertile period. Our observed association is consistent with published research which indicates that women using natural family planning methods usually abstain from unprotected sexual intercourse during their fertile days to avoid unintended pregnancy [16, 18]. Nevertheless, the success of periodic abstinence also depends on the woman’s ability to accurately determine their fertile period and to abstain from unprotected sexual intercourse [17, 18, 40].

Although the findings of the present study are encouraging, there are a few limitations that need to be highlighted. Firstly, the cross-sectional nature of the study makes it difficult to determine the direction of causality. Secondly, the findings of this study cannot be generalized given that a convenience sampling technique was used to recruit female students who participated in the parent study. Nevertheless, this study has generated data on fertility awareness knowledge in a country where there was very little information available. The study has also contributed to new ideas and knowledge on the relationship between fertility awareness knowledge, and contraceptive methods used by FUS.

Our findings have programmatic and policy implications. Firstly, it underscores the need for FUS to be targeted with high-quality reproductive health information to improve their fertility knowledge. It also suggests the need for integrating menstrual health education into sexual and reproductive programs for FUS and other young women. Targeted fertility awareness educational interventions are needed to improve the fertility awareness knowledge of FUS. This could be provided by health care personnel working at the on-campus, university health services. These on-campus health services could organize regular reproductive health sessions where fertility awareness and menstrual cycle education is shared with FUS to help them improve their fertility awareness knowledge. Relevant materials to educate FUS on their fertility and menstrual health could also be provided through leaflets, and brochures which are distributed in university campuses. Furthermore, FUS could also be targeted with fertility-related information via social media platforms to help raise their fertility awareness to prevent unwanted pregnancy. Further studies should examine the relationship between low fertility awareness knowledge and emergency contraceptive use.

## Conclusions

Our study found that the majority of the sexually active FUS intend to have children in the future. Although fertility awareness knowledge was suboptimal there was a high rate of contraceptive use. There was a statistically significant association between fertility awareness knowledge and the use of male condoms, withdrawal, and periodic abstinence. The gap in fertility awareness knowledge identified in this study underscores the need for FUS to be targeted with fertility awareness education through social media platforms and other reproductive health education programs organized by on-campus health personnel. These interventions will help improve their fertility awareness knowledge and guide them in making informed decisions on their contraceptive behaviour.

## Supporting information

S1 Dataset This is the dataset used in the analysis. (XLS)
